# β-catenin stabilization enhances *SS18-SSX2*-driven synovial sarcomagenesis and blocks the mesenchymal to epithelial transition

**DOI:** 10.18632/oncotarget.4283

**Published:** 2015-06-08

**Authors:** Jared J. Barrott, Benjamin E. Illum, Huifeng Jin, Ju-Fen Zhu, Tim Mosbruger, Michael J. Monument, Kyllie Smith-Fry, Matthew G. Cable, Yanliang Wang, Allie H. Grossmann, Mario R. Capecchi, Kevin B. Jones

**Affiliations:** ^1^ Department of Orthopaedics and Huntsman Cancer Institute, University of Utah School of Medicine, Salt Lake City, UT 84112, USA; ^2^ Department of Bioinformatics and Huntsman Cancer Institute, University of Utah School of Medicine, Salt Lake City, UT 84112, USA; ^3^ Department of Pathology and ARUP Laboratories, University of Utah School of Medicine, Salt Lake City, UT 84112, USA; ^4^ Department of Human Genetics, University of Utah School of Medicine, Salt Lake City, UT 84112, USA

**Keywords:** epithelial-mesenchymal transition, translocation, Wnt-signaling, mouse genetic model

## Abstract

β-catenin is a master regulator in the cellular biology of development and neoplasia. Its dysregulation is implicated as a driver of colorectal carcinogenesis and the epithelial-mesenchymal transition in other cancers. Nuclear β-catenin staining is a poor prognostic sign in synovial sarcoma, the most common soft-tissue sarcoma in adolescents and young adults. We show through genetic experiments in a mouse model that expression of a stabilized form of β-catenin greatly enhances synovial sarcomagenesis. Stabilization of β-catenin enables a stem-cell phenotype in synovial sarcoma cells, specifically blocking epithelial differentiation and driving invasion. β-catenin achieves its reprogramming in part by upregulating transcription of TCF/LEF target genes. Even though synovial sarcoma is primarily a mesenchymal neoplasm, its progression towards a more aggressive and invasive phenotype parallels the epithelial-mesenchymal transition observed in epithelial cancers, where β-catenin's transcriptional contribution includes blocking epithelial differentiation.

## INTRODUCTION

Synovial sarcoma (SS) is the most common soft-tissue sarcoma diagnosed during adolescence and young adulthood [1]. Clinically recalcitrant to traditional cytotoxic chemotherapy in most cases, once metastatic, SS is usually fatal. This leads to early demise in nearly half of its victims, overall. SSs bear a t(X;18) translocation, producing one of three possible *SS18-SSX* fusion oncogenes [2]. Expression of the complementary DNA (cDNA) for *SS18-SSX2* in specific cell lineages induces a completely penetrant and faithful recapitulation of SS in the mouse [3]. Both human and induced mouse SSs demonstrate a unique and striking character that includes both epithelial and mesenchymal differentiation. Despite mesenchymal origins, some SSs develop epithelial glandular structures, termed the biphasic histologic type and suggestive of a mesenchymal-epithelial transition (MET).

β-catenin functions as an epigenetic transcriptional cofactor in development and oncogenesis and drives the epithelial-mesenchymal transition in cancer [4]. The Axin-APC-GSK3β complex regulates β-catenin levels through phosphorylation, targeting it for ubiquitination and proteasomal degradation [5]. Canonical Wnt signal transduction, APC inactivating mutations, or mutations in the third exon of β-catenin each result in the prevention of this phosphorylation, stabilizing β-catenin and driving it to the nucleus.

β-catenin stabilizing mutations and APC silencing mutations have been identified in SSs [6, 7]. Nuclear immunohistochemical β-catenin staining, reported in 30–70% of SSs, associates with poor prognosis [7, 8]. Genetic disruption of β-catenin blunts synovial sarcomagenesis driven by *SS18-SSX2* in the mouse *Myf5Cre* lineage, suggesting near necessity of Wnt/β-catenin signaling to the transformation of that lineage [9]. We investigated the impact of β-catenin stabilization in synovial sarcomagenesis in the mouse model.

## RESULTS

### β-catenin stabilization enhances SS18- SSX2-driven tumorigenesis

**Figure 1:**
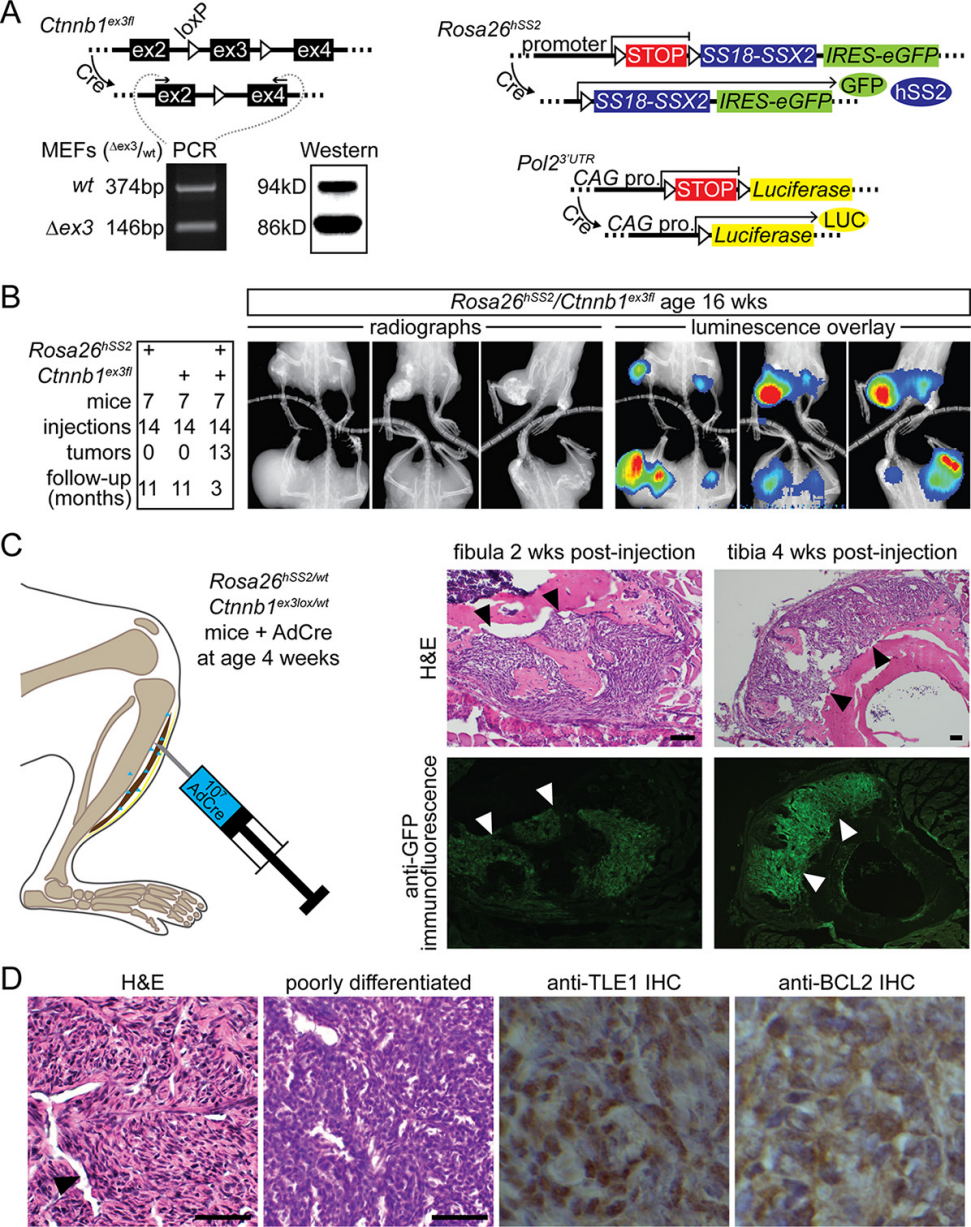
β-catenin stabilization enhances SS18-SSX2-driven tumorigenesis **A.** Schematics represent the conditional alleles for expression of *SS18-SSX2* from the *Rosa26* locus, *Luciferase* from a *CAG* promoter in the 3′ untranslated region of the *RNA polymerase 2* locus, and removal of the third exon in *Ctnnb1*. Total RNA and protein obtained from mouse embryonic fibroblasts and analyzed by RT-PCR and Western demonstrate relative stabilization at the protein level of the β-catenin isoform resulting from removal of the third exon (Δex3). **B.** Tabulated tumor formation in 3 groups of mice bearing *Rosa26^hSS2^* and/or *Ctnnb1^ex3fl^*, injected at 4 weeks with AdCre. Radiographs and luciferase luminescence overlay images of these tumors 12 weeks after AdCre injection. **C.** Localized injection of AdCre into the pretibial soft-tissues leads to early tumor development outside the bone and reactive osteoid formation detectable at 2 and 4 weeks after the injection. (Arrows indicate the normal position of periosteum, here replaced by tumor in each example.) **D.** Representative histologic sections from the same cohort of mice stained with H&E demonstrate the classic SS histopathology, including hemangiopericytomatous vascular spaces (black arrow) and poorly differentiated areas with cells dominated by large nuclei and scant cytoplasm. Immunohistochemical stains demonstrate abundant nuclear TLE1 and cytoplasmic BCL2 in these tumors. (Magnification bars are 40 μm. Immuno-photomicrograph panels are 40 μm square.)

The *Ctnnb1^ex3f l^* allele has *loxP* sites flanking the third exon [10]. Cre-mediated recombination yields *Ctnnb1^Δex3^*, which expresses a shortened and stabilized β-catenin with normal trans-activation capabilities and a predilection for the nucleus [10]. Fibroblasts from *Rosa26^hSS2^/_wt_*; *Ctnnb1^ex3fl^/_wt_* embryos exposed to Cre-recombinase in culture demonstrated increased protein presence of the smaller, stabilized, Δex3fl form, despite equivalent transcription (Figure 1A). *Myf5-Cre*; *Ctnnb1^ex3fl^* mice were not viable (data not shown), necessitating another strategy for initiating synovial sarcomagenesis. First, we injected an adeno-associated virus (AdCre) into the bilateral hindlimbs of 7 four-week-old mice of each of 3 genotypes, *Rosa26^hSS2^/_wt_*; *Ctnnb1^wt^/_wt_*, *Rosa26^wt^/_wt_*; *Ctnnb1^ex3fl^/_wt_*, and *Rosa26^hSS2^/_wt_*; *Ctnnb1^ex3fl^/_wt_*. All mice carried a luciferase reporter allele (Figure 1A). By 1 year, neither *Rosa26^hSS2^*, nor *Ctnnb1^ex3f l^* alone produced tumors (Figure 1B). Nearly all injections into combination genotype mice produced massive tumors by 3 months (Figure 1B).

### Stabilization of β-catenin drives an invasive tumor phenotype

Tumors that developed following AdCre injections involved the entire hindlimb and destroyed the skeletal architecture in many instances (Figure 1B). To differentiate invasion following extra-skeletal tumorigenesis from intra-osseous tumorigenesis, we induced tumors from spatially restricted injection of AdCre into the tibialis anterior muscle (Figure 1C), serially sectioning limbs 2, 4 and 8 weeks after injection. Tumors were consistently adjacent to, but never directly involving the bone, at the first two time points (Figure 1C). Abundant reactive bone formed around developing tumors, but invasion only followed, between 4 and 8 weeks after AdCre injection. This suggested that invasion, rather than intra-osseous tumorigenesis, led to the destruction of skeletal elements.

### SS18-SSX2 expression concomitant to β-catenin stabilization produces synovial sarcomas

Tumors that formed in AdCre-injected *Rosa26^hSS2^/_wt_*; *Ctnnb1^ex3fl^/_wt_* mice demonstrated classic SS histologic features: short spindle cells with intervening staghorn-style hemangiopericytomatous vascularity (Figure 1D). Most also included areas with more prominent nuclei and scant cytoplasm, fitting the most aggressive histologic group of SSs, termed poorly differentiated SS (Figure 1D). By immunohistochemistry, tumors stained for classic diagnostic markers, BCL2 and TLE1 (Figure 1D) [11].

**Figure 2:**
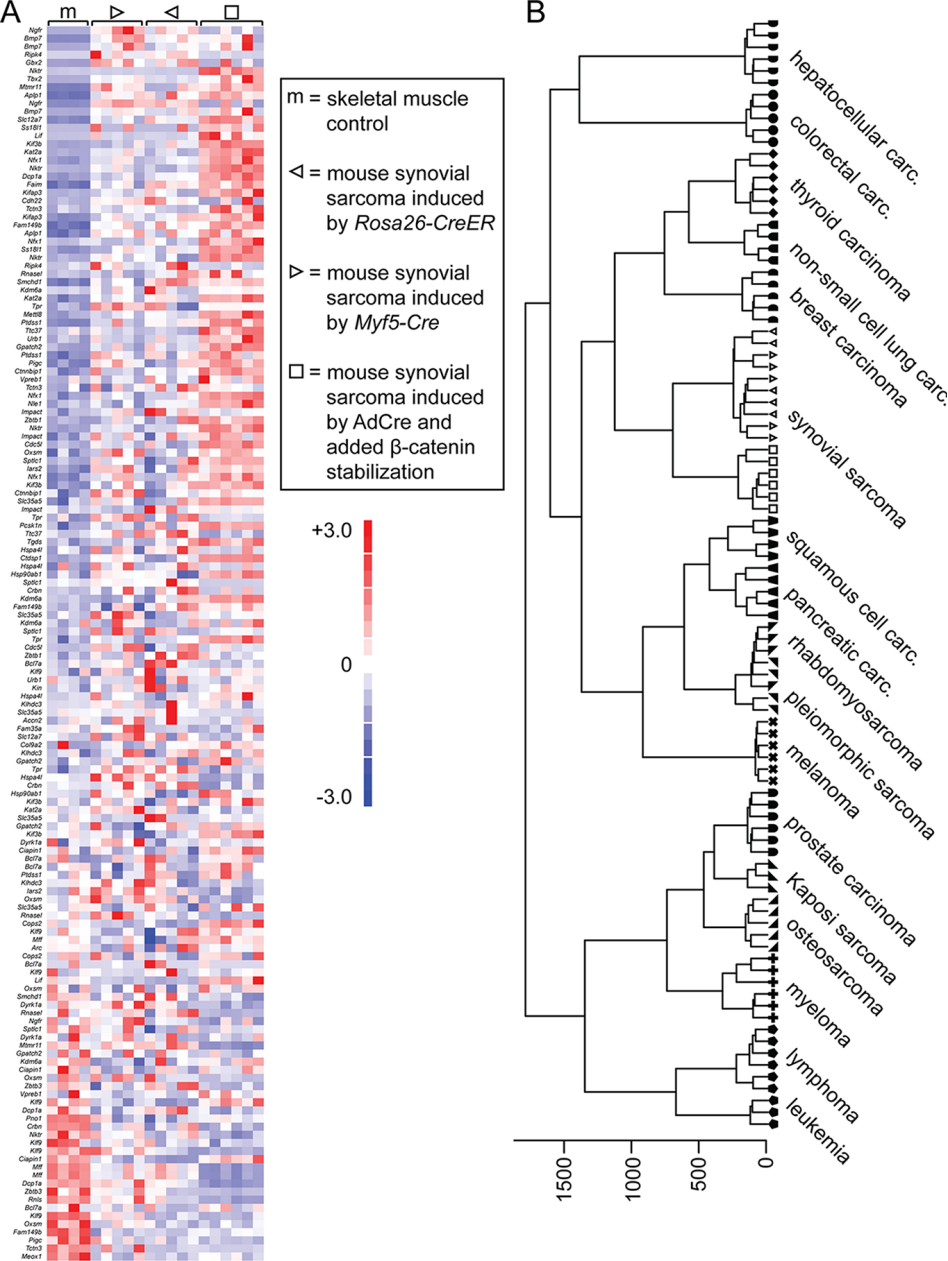
Tumors developing in mice with stabilized β-catenin are synovial sarcomas by transcriptome **A.** Heat map of a list of genes previously determined to be enriched in human and mouse SSs, comparing control skeletal muscle (noted by m) to two mouse models of SS without conditional genetic stabilization of *Ctnnb1* (noted by triangles) and the current model wherein β-catenin is stabilized concurrent to expression of *SS18-SSX2*, induced by AdCre injection into the hind limb soft-tissues (noted by squares). **B.** Unsupervised hierarchical clustering of these three models of mouse SS, compared to 78 other genetically-induced mouse tumor models.

Microarray analysis of gene expression enabled comparison to extant data from prior models. Tumors from AdCre injected *Rosa26^hSS2^/_wt_*; *Ctnnb1^ex3f l^/_wt_* mice demonstrated strong expression of a gene signature previously shown to be enriched in human SSs [3], similar to tumors arising in *Myf5-Cre*; *Rosa26^hSS2^/_wt_* and *Rosa26^hSS2^/_CreER_* [12] mice, which lack genetic stabilization of β-catenin (Figure 2A). By gene set enrichment analysis, the normalized enrichment score (NES) for β-catenin stabilized tumors was 1.51 and false discovery rate (FDR) *q*-value 0.025. In comparison, for the *Myf5-Cre*; *Rosa26^hSS2^/_wt_* tumors from which the gene set was defined, the NES was 1.46 and FDR *q*-value 0.043. Generally, the NES measures the degree to which the gene set is enriched (or preferentially represented among the highly expressed genes) in the test dataset, normalized for the size of the gene set itself. Stringent *q*-values are less than 0.05.

A panel of mouse cancer models (Supplemental Table 1) with expression data from the same platform available on the Gene Expression Omnibus (GEO) were used for an unsupervised hierarchical clustering, which placed mouse SSs—with or without stabilized β-catenin—adjacent, but not intermingled on the cluster (Figure 2B). These data suggested that stabilization of β-catenin had not suppressed the SS identity, but that the tumors represent a distinct class of SSs from those initiated without β-catenin stabilization.

### TATCre initiates tumorigenesis from SS18-SSX2 with or without β-catenin stabilization

**Figure 3:**
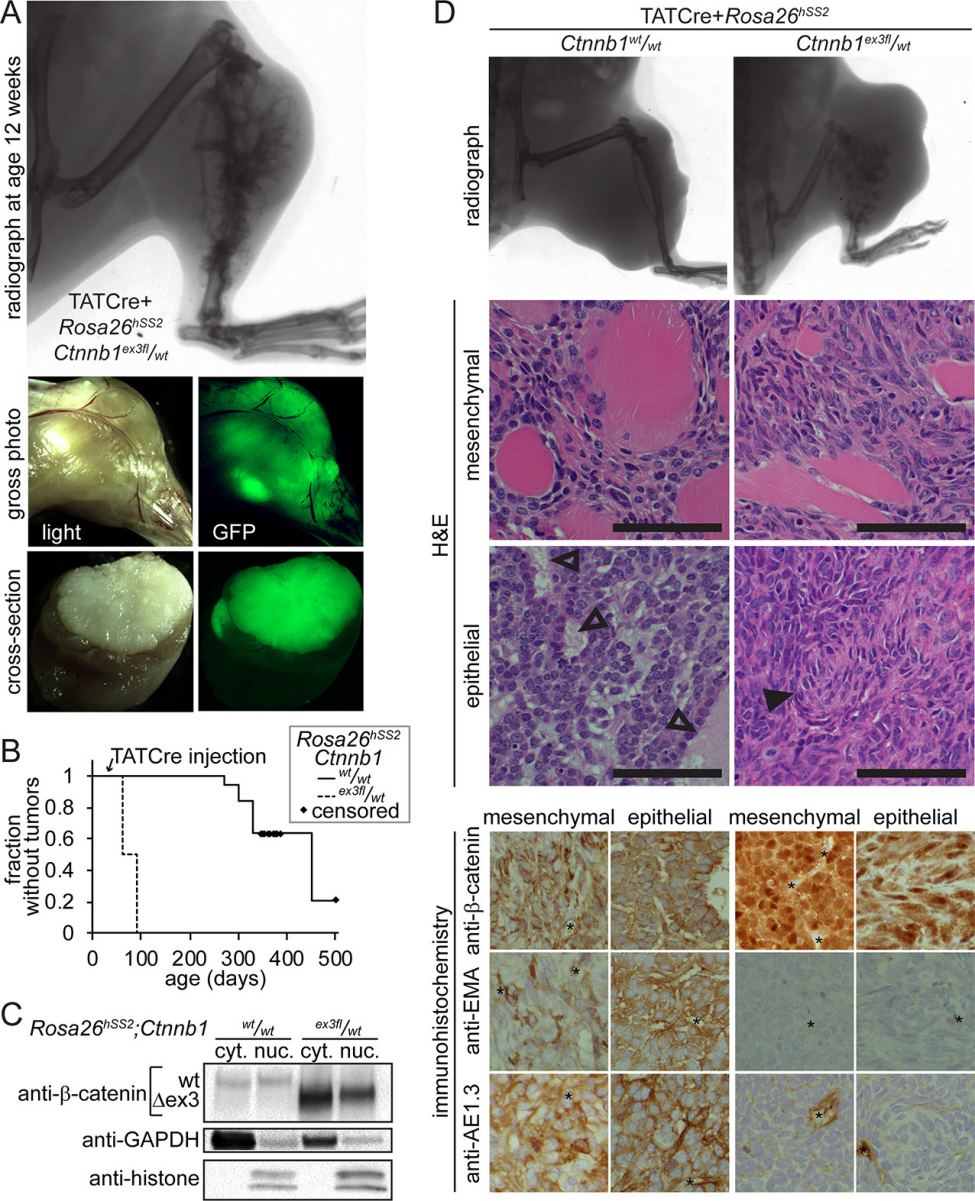
Initiation of tumors with injected TATCre enables comparison of SS18-SSX2-induced tumors with and with genetic stabilization of β-catenin **A.** Radiograph, gross and cross sectional photographs each with light and GFP fluorescence demonstrate the typical tumor that has developed by 8 weeks after TATCre injection into *Rosa26^hSS2^/_wt_*; *Ctnnb1^ex3fl^/_wt_* mice at age 4 weeks. **B.** Kaplan-Meier plot of the relative latency to tumorigenesis following TATCre injection at age 4 weeks into *Rosa26^hSS2^/_wt_* mice with (*n* = 16) or without (*n* = 19) an *ex3f l* allele of *Ctnnb1*. **C.** Westerns of cytoplasmic (cyt.) and nuclear (nuc.) protein fractions from tumors demonstrate degradation to below detectable levels of full length β-catenin in tumors heterozygous for the stabilized, Δex3 form, suggesting feedback activation. **D.** Comparison of radiographic evidence of skeletal element invasion, muscle invasion by monophasic areas of tumor cells, the maximal epithelial differentiation, and immunophenotype of both monophasic and biphasic areas of TATCre-induced *Rosa26^hSS2^/_wt_* tumors with or without stabilized β-catenin. (Magnification bars are 50 μm. Immunohistochemistry photomicrographs are 50 μm-square panels. Open arrows indicate epithelial glands. Filled arrows indicate swirls of mesenchymal appearing cells, the structure most similar to glands that formed in tumors with stabilized β-catenin. * indicates position of vessel.)

The transcriptomic differences from prior models and aggressive growth pattern of AdCre-induced *Rosa26^hSS2^/_wt_*; *Ctnnb1^ex3f l^/_wt_* tumors could not be strictly attributed to the β-catenin stabilization alone, given the distinct tumor initiation method. Absence of control tumors developing within a year following AdCre mice limited opportunities to compare tumor phenotypes and transcriptomes. We next injected the protein TATCre [13] into the tibialis anterior muscle and underlying periosteum. TATCre generated rapid, fully-penetrant tumorigenesis following each injection into *Rosa26^hSS2^/_wt_*; *Ctnnb1^ex3fl^/_wt_* mice (Figure 3A). *Rosa26^hSS2^/_wt_*; *Ctnnb1^wt^/_wt_* mice also developed tumors, at longer latency and lower prevalence (Figure 3B). Increasing the number of control group mice enabled collection of comparable groups of TATCre-induced tumors.

### β-catenin drives an EMT in synovial sarcoma

The *Ctnnb1^ex3fl^* allele led to increased nuclear β-catenin and reduction of the non-stabilized form to an undetectable level, suggesting upregulated feedback (Figure 3C). Even when sufficient time led to large-sized tumors, *Rosa26^hSS2^/_wt_*; *Ctnnb1^wt^/_wt_* tumors never invaded and destroyed adjacent skeletal elements as *Rosa26^hSS2^/_wt_*; *Ctnnb1^ex3fl^/_wt_* tumors did consistently (Figure 3D). Biphasic histologic features developed exclusively in the absence of genetically stabilized β-catenin. Tumors formed in combined genotype mice developed no morphologies more epithelial than swirls of mesenchymal-appearing cells and demonstrated reduced expression of epithelial markers, EMA and pan-cytokeratin (Figure 3D).

**Figure 4:**
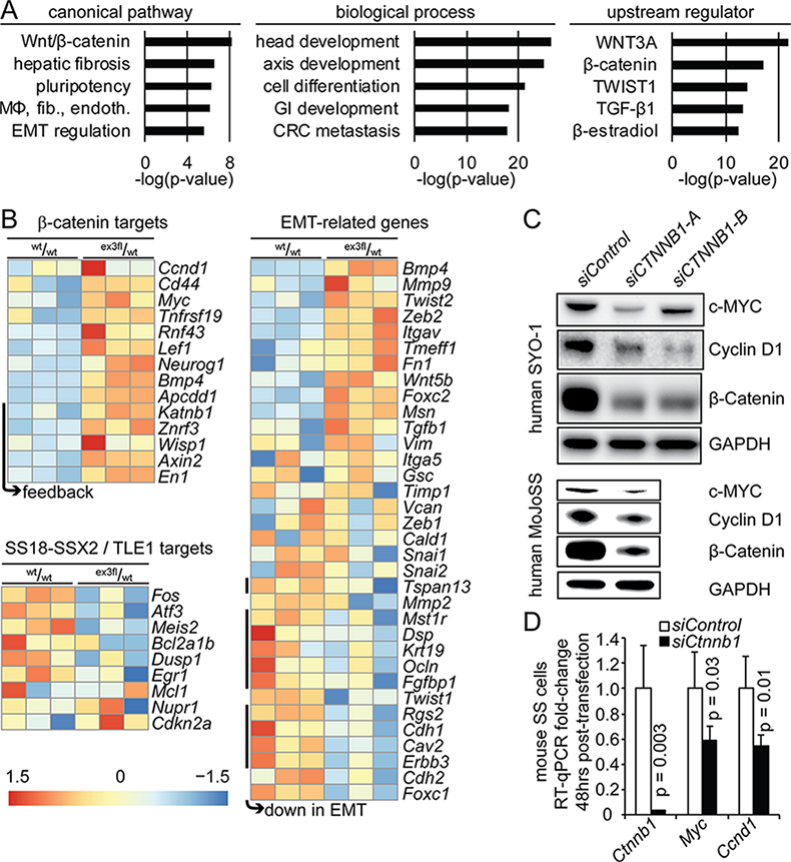
Stabilization of β-catenin drives an EMT transcriptional signature and increases expression of TCF/LEF target genes **A.** Chart of the log of the *p*-values for the top 5 canonical pathways, biological processes, and upstream regulators from ingenuity pathway analysis on the genes that were differentially expressed between TATCre-initiated *Rosa26^hSS2^* tumors with and without stabilized β-catenin. (MΦ, fib., endoth. indicates the macrophages, fibroblasts and endothelial cells activated in rheumatoid arthritis. GI indicates gastrointestinal. CRC indicates colorectal carcinoma.) **B.** Heat maps of differentially expressed β-catenin target genes, target genes of *SS18-SSX2*-mediated transcriptional repression via TLE1, and genes related to the epithelial-to-mesenchymal transition (EMT). **C.** Western blots from two human SS cell lines 72 hours after transfection with either control or anti-*CTNNB1* small interfering RNA (siRNA) molecules. **D.** Chart of fold-expression by RT-qPCR of *Myc* and *Ccnd1* 48 hours following siRNA against *Ctnnb1* in mouse synovial sarcoma cells.

RNA from TATCre-induced *Rosa26^hSS2^* tumors with either *Ctnnb1* genotype was sequenced for detailed transcriptome comparison. Importantly, the specific *Ctnnb1^wt^/_wt_* tumors sequenced were also of monophasic histology, so as not to bias the differences with frankly epithelial differentiation in the control group. Nonetheless, differential expression highlighted not only Wnt/β-catenin pathway target genes, but also a deepening of the TLE1-driven repression of *SS18-SSX* target genes and derangement of EMT-related genes (Figure 4A–4B).

To test the relationship between β-catenin and TCF/LEF target genes highlighted in the comparison of tumors extends to SS cell lines, we knocked down *CTNNB1/Ctnnb1* in human and mouse SS cell lines by siRNA. Both c-MYC and Cyclin-D1 reduced with β-catenin (Figure 4C–4D).

## DISCUSSION

We have shown that stabilization of β-catenin powerfully enhances synovial sarcomagenesis driven by *SS18-SSX2*, inducing an aggressively invasive tumor phenotype. The lack of biphasic histologic features in TATCre- or AdCre-induced *Rosa26^hSS2^/_wt_*; *Ctnnb1^ex3f l^/_wt_* tumors does not make them any less comparable to human SS, as the majority of human SSs also lack this biphasic histopathology, but it does suggest that β-catenin stabilization blocks epithelial differentiation. While metastasis itself would be an ideal phenotype to measure in these mice with stabilized β-catenin, the rapid tumorigenesis leads to morbidity from primary tumors, necessitating euthanasia too quickly to permit metastases to develop at detectable levels. Even so, these data explain in part the previously reported correlation between nuclear β-catenin staining and poor prognosis in SS [7, 8]. The mouse data also explain the role played by *CTNNB1* stabilizing and *APC* inactivating mutations in up to 20 percent of human SSs [6, 7]. Notably, all such described tumors with these mutations were monophasic fibrous SSs, lacking epithelial differentiation. Therefore, β-catenin blocks epithelial differentiation, or an MET in this mesenchymal neoplasm. Because even a formal EMT as described in epithelial carcinomas derives primarily from a block to epithelial differentiation, one may cautiously invoke the term EMT to describe this block of the epithelial transition in SS.

Of special interest was the slightly enhanced suppression of a number of target genes known to be transcriptionally down-regulated by the fusion oncoprotein. This was paradoxical, given the fact that TLE1 mediates SS18-SSX target gene suppression [14] and that β-catenin's primary transcriptional function is to antagonize TLE1 at TCF/LEF-bound promoters [15]. Knowing these relationships, one might predict that β-catenin would relieve suppression of these target genes by antagonizing TLE1 binding. One explanation for the observed enhanced suppression of these SS18-SSX target genes is that the antagonism of TLE1 by an increase in nuclear β-catenin liberates TLE1 from TCF/LEF-bound promoters. This increased pool of TLE1 may form more complexes with SS18-SSX2, thus enhancing the suppression of its target genes. While these data derive from an analysis of only 6 tumors, and certainly do not prove this alternate explanation, they do reject the likelihood of an antagonism between β-catenin and TLE1 with regard to the latter's interaction with the SS18-SSX fusion oncoprotein.

SS has been termed a stem cell malignancy [16]. Some degree of stemness may be necessary to enable SS18-SSX-driven transformation. This hypothesis is highlighted by the nine month delay in TATCre-induced tumor formation in mice with *Rosa26^hSS2^/_wt_*; *Ctnnb1^wl^/_wt_* compared to mice with *Rosa26^hSS2^/_wt_*; *Ctnnb1^ex3fl^/_wt_*. These data must be compared to earlier reports of the *Rosa26^hSS2^/_wt_* allele activated in the *Myf5Cre* lineage, which results in much faster tumorigenesis, usually before 6 months' age [3]. Why *SS18-SSX2* expression is more rapidly oncogenic in the *Myf5Cre* lineage than following TATCre injection may derive in part from the simply stochastic principle that an entire lineage throughout the mouse begins expressing the fusion oncogene following *Myf5Cre* expression, as opposed to one small area of a limb after TATCre injection. Certainly, the other model of Cre-mediated induction in a few scattered cells throughout a mouse has a latency to tumorigenesis closer to that following TATCre [12]. However, there is also likely some contribution from characteristics of the originating cells in the *Myf5Cre* lineage. *Myf5Cre* initiates tumors during embryogenesis, when the *Myf5Cre* lineage retains pluripotency and many stem-like properties. Very likely, these embryonic mesenchymal progenitor cells have strong native Wnt/β-catenin signaling, which aids and abets SS18-SSX-driven transformation. Wnt/β-catenin signaling has long been known to be heavily involved in programming stemness in mesenchyme.

The dramatic enhancement of synovial sarcomagenesis from the stabilization of β-catenin suggests that Wnt signaling can help a cell tolerate the presence of the SS18-SSX2 fusion oncoprotein. If a cell requires no “second hit” more drastic than stabilization of β-catenin to render synovial sarcomagenesis efficient, it may achieve that stabilization in a variety of ways. APC inactivating and β-catenin stabilizing mutations have been described in SS [6, 7], but many other genetic, epigenetic, paracrine, and microenvironmental forces converge upon β-catenin stabilization and may provide alternate means of enabling SS18-SSX-driven oncogenesis. It is noteworthy that induced SS18-SSX-driven synovial sarcomas in mice demonstrate approximately 50% prevalence of the biphasic histologic subtype in tumors > 1 cm, whereas this subtype is only present in approximately 25% of human SSs. Alterations in Wnt/β-catenin signaling, acquired during the longer and larger growth of human tumors may explain the relative suppression of this phenotype in human tumors.

## MATERIALS AND METHODS

### Mice

Mouse experiments were conducted with the approval of the institutional animal care committee in accordance with legal and ethical standards. The previously described *Rosa26^hSS2^* [3], *Ctnnb1^ex3fl^* [10], and *Myf5-Cre* [3] mice were maintained on a mixed strain background, C57BL/6 and SvJ. Littermate controls were used for experimental comparisons. Genotyping primer sequences are in the supplemental methods.

AdCre (Gene Transfer Vector Core Facility, University of Iowa, Iowa City, IA, USA) injections included 10^7^ units in 100 μL saline. TATCre injections were 30 μL at 100 μM.

Radiographs were obtained with a Kodak Carestream 4000ProFx (Carestream Health, Inc., Rochester, NY, USA), brightfield and fluorescent images with a Leica AF6000 dissecting microscope (Leica Microsystems, Wetzlar, Germany), and light photomicrographs with an Olympus BX43 microscope and DP26 camera (Olympus America, Center Valley, PA, USA).

### Cell lines

The human SYO-1 cell line [17], received from Torsten Nielsen at the University of British Columbia, was maintained in Dulbecco's modified eagle medium (DMEM) with 10% fetal bovine serum (FBS), and validated every 3 months by expression of and dependence (siRNA) on *SS18-SSX2*. The MoJoSS cell line, derived by dissociation and culture of a metastatic focus of monophasic SS in a lymph node, expresses *SS18-SSX1*, was maintained in DMEM with 20% FBS. The mouse cell line was derived from tumors that arose in a *Myf5-Cre*; *Rosa26^hSS2^* mouse, was maintained in DMEM with 10% FBS. Validation of both developed cell lines also includes fusion gene expression and testing of dependence by siRNA, every 3 months in culture. SiRNA against mouse *Ctnnb1* (SASI_Mm01_00161715; Sigma) and human *CTNNB1* (A = SASI_Hs01_00117960; B = SASI_Hs01_00117958; Sigma) were applied with Lipofectamine RNAiMax (Life Technologies, Invitrogen), using 3–4 μL of lipid reagent and 12–50 nM siRNA.

### Transcriptome analyses

Total RNA was isolated with RNeasy mini kit (Qiagen, Valencia, CA, USA). Complementary DNAs were generated using Maxima first-strand (Fermentas Life Sciences). Quantitative PCR used the SYBR Green Master Mix (Applied Biosystems, Life Technologies) and an Applied Biosystems 7900HT. Primer sequences are in the supplemental methods.

Genome-wide expression profiling of AdCre tumors followed reverse transcription by One-Cycle Target Labeling with hybridization on the Mouse 430 2.0 Gene Chip (Affymetrix, Santa Clara, CA, USA). Microarray data were analyzed using DChip [18] and GeneSifter (Geospiza, Inc.). Gene set enrichment analysis used Broad Institute software [19].

For transcriptome sequencing of TATCre tumors, RNA was prepared using the Illumina TruSeq RNA kit (Illumina, Inc., San Diego, California), checked with the Agilent Bioanalyzer RNA 6000 chip (Agilent Technologies, Santa Clara, California), captured using the RiboZero method (Illumina), and 50-cycle end-read sequenced on an Illumina HiSeq 2000.

Reference fasta files were generated by combining the chromosome sequences from mm10 with all possible splice junction sequences, which were generated with USeq (v8.8.8) MakeTranscriptome using a radius of 46 and annotated with Ensembl transcripts (build 74) from the UCSC browser. Reads were aligned with Novoalign (v2.08.01), allowing up to 50 alignments per read. USeq's SamTranscriptomeParser selected the best alignment for each and converted the coordinates of reads aligning to splices back to genomic space. Differential gene expression was measured using USeq's DefinedRegionDifferentialSeq. Briefly, the number of reads aligned to each gene were calculated, then normalized in DESeq2 [20]. R package ‘pheatmap’ (v1.0.2) generated heatmaps. Log2(FPKM) values were centered and scaled by gene.

### Protein analysis

Protein was isolated using the M-PER reagent (Thermo Scientific). For cytoplasmic and nuclear fractions, tumors were homogenized to a single cell suspension. Cells were incubated 10 min in a mild lysis buffer and the cytoplasmic fraction was collected. The pellet was washed once in nuclear wash buffer, then purified over a 60% Percoll/sucrose gradient. The nuclear fraction was collected and lysed in nuclear lysis buffer on ice for 10 min. Both fractions were clarified at 14,000 rpm for 10 min before loading 25 μg/sample for gel electrophoresis and transfer to PVDF membrane. Western blots were probed with antibodies for β-catenin, c-MYC, Cyclin D1, H3, and GAPDH. Supplemental methods contains an antibody chart and buffer compositions.

### Histology

Tissues were fixed in 4% paraformaldehyde overnight, decalcified (if including bone) in 14% ethylenediaminetetraacetic acid at pH 7.4 for two weeks at 4°C, and embedded in paraffin. Immunohistochemistry for mouse TLE1, BCL2, pan-cytokeratin, and epithelial membrane antigen were performed using IgG-horse radish peroxidase and hematoxylin counterstain.

## SUPPLEMENTARY DATA TABLES



## References

[1] Herzog CE (2005). Overview of sarcomas in the adolescent and young adult population. J Pediatr Hematol Oncol.

[2] Ladanyi M, Antonescu CR, Leung DH, Woodruff JM, Kawai A, Healey JH, Brennan MF, Bridge JA, Neff JR, Barr FG, Goldsmith JD, Brooks JS, Goldblum JR, Ali SZ, Shipley J, Cooper CS, Fisher C, Skytting B, Larsson O (2002). Impact of SYT-SSX fusion type on the clinical behavior of synovial sarcoma: a multi-institutional retrospective study of 243 patients. Cancer Res.

[3] Haldar M, Hancock JD, Coffin CM, Lessnick SL, Capecchi MR (2007). A conditional mouse model of synovial sarcoma: insights into a myogenic origin. Cancer cell.

[4] Moon RT, Kohn AD, De Ferrari GV, Kaykas A (2004). WNT and beta-catenin signalling: diseases and therapies. Nat Rev Genet.

[5] Liu C, Li Y, Semenov M, Han C, Baeg GH, Tan Y, Zhang Z, Lin X, He X (2002). Control of beta-catenin phosphorylation/degradation by a dual-kinase mechanism. Cell.

[6] Saito T, Oda Y, Sakamoto A, Kawaguchi K, Tanaka K, Matsuda S, Tamiya S, Iwamoto Y, Tsuneyoshi M (2002). APC mutations in synovial sarcoma. J Pathol.

[7] Saito T, Oda Y, Sakamoto A, Tamiya S, Kinukawa N, Hayashi K, Iwamoto Y, Tsuneyoshi M (2000). Prognostic value of the preserved expression of the E-cadherin and catenin families of adhesion molecules and of beta-catenin mutations in synovial sarcoma. J Pathol.

[8] Hasegawa T, Yokoyama R, Matsuno Y, Shimoda T, Hirohashi S (2001). Prognostic significance of histologic grade and nuclear expression of beta-catenin in synovial sarcoma. Hum Pathol.

[9] Barham W, Frump AL, Sherrill TP, Garcia CB, Saito-Diaz K, VanSaun MN, Fingleton B, Gleaves L, Orton D, Capecchi MR, Blackwell TS, Lee E, Yull F, Eid JE (2013). Targeting the Wnt pathway in synovial sarcoma models. Cancer discovery.

[10] Harada N, Tamai Y, Ishikawa T, Sauer B, Takaku K, Oshima M, Taketo MM (1999). Intestinal polyposis in mice with a dominant stable mutation of the beta-catenin gene. EMBO J.

[11] Jagdis A, Rubin BP, Tubbs RR, Pacheco M, Nielsen TO (2009). Prospective evaluation of TLE1 as a diagnostic immunohistochemical marker in synovial sarcoma. Am J Surg Pathol.

[12] Haldar M, Hedberg ML, Hockin MF, Capecchi MR (2009). A CreER-based random induction strategy for modeling translocation-associated sarcomas in mice. Cancer research.

[13] Straessler KM, Jones KB, Hu H, Jin H, van de Rijn M, Capecchi MR (2013). Modeling clear cell sarcomagenesis in the mouse: cell of origin differentiation state impacts tumor characteristics. Cancer Cell.

[14] Su L, Sampaio AV, Jones KB, Pacheco M, Goytain A, Lin S, Poulin N, Yi L, Rossi FM, Kast J, Capecchi MR, Underhill TM, Nielsen TO (2012). Deconstruction of the SS18-SSX fusion oncoprotein complex: insights into disease etiology and therapeutics. Cancer cell.

[15] Daniels DL, Weis WI (2005). Beta-catenin directly displaces Groucho/TLE repressors from Tcf/Lef in Wnt-mediated transcription activation. Nature structural & molecular biology.

[16] Naka N, Takenaka S, Araki N, Miwa T, Hashimoto N, Yoshioka K, Joyama S, Hamada K, Tsukamoto Y, Tomita Y, Ueda T, Yoshikawa H, Itoh K (2010). Synovial sarcoma is a stem cell malignancy. Stem Cells.

[17] Kawai A, Naito N, Yoshida A, Morimoto Y, Ouchida M, Shimizu K, Beppu Y (2004). Establishment and characterization of a biphasic synovial sarcoma cell line, SYO-1. Cancer Lett.

[18] Li C, Hung Wong W (2001). Model-based analysis of oligonucleotide arrays: model validation, design issues and standard error application. Genome biology.

[19] Subramanian A, Tamayo P, Mootha VK, Mukherjee S, Ebert BL, Gillette MA, Paulovich A, Pomeroy SL, Golub TR, Lander ES, Mesirov JP (2005). Gene set enrichment analysis: a knowledge-based approach for interpreting genome-wide expression profiles. Proc Natl Acad Sci U S A.

[20] Love MI, Huber W, Anders S (2014). Moderated estimation of fold change and dispersion for RNA-seq data with DESeq2. Genome biology.

