# Supplementary material for: β-catenin stabilization enhances SS18-SSX2-driven synovial sarcomagenesis and blocks the mesenchymal to epithelial transition
Source: Oncotarget. 2015 Jun 8;6(26):22758–66. doi: 10.18632/oncotarget.4283 (PMC4673197; doi:10.18632/oncotarget.4283)
Supplement: Supplementary file 1 [file oncotarget-06-22758-s001.pdf]

SUPPLEMENTARY DATA

Supplementary Table S1. Genotyping primer sequences and amplicon sizes

gene	species	primer sequence (5'-3')	cDNA amplicon	gDNA amplicon
Cre		GGATTTCCGTCTCTGGTGTAGC<br>ACCATTGCCCCTGTTTCACTATC		337
Ctnnb1-ex3fl	mouse	GTTAGAAAGGCAGCCAAGGAG<br>CAATGGCTACTCAAGGTTTGTG		ex3lox: 450<br>wt: 220
Rosa26	mouse	GTTATCAGTAAGGGAGCTGCAGTGG<br>AAGACCGCGAAGAGTTTGTCCTC<br>GGCGGATCACAAGCAATAATAACC		*hSS2*/*LacZ*/<br>*CreER*: 302<br>wt: 415
Myf5-Cre	mouse	ACCCTCCAGCTCCAGACTTATC<br>CCCTGTAATGGATTCCAAGCTG<br>AAAGACCCCTAGGAATGCTC		IRES-Cre: 594<br>wt: 451
Ctnnb1 exon2 to exon4	mouse	GCGTGGACAATGGCTACTCAA<br>ACATTAGTGGGATGAGCAGCG	Δex3: 146<br>ex3fl or wt:<br>374	Δex3: 350<br>wt: 806

Supplementary Table S2. RT-qPCR Primer sequences and amplicon sizes

gene	species	Primer sequences (5' to 3')	cDNA amplicon size
Ctnnb1	mouse	ATGGAGCCGGACAGAAAAGC<br>CTTGCCACTCAGGGAAGGA	108
Myc	mouse	ATGCCCCTCAACGTGAACTTC<br>CGCAACATAGGATGGAGAGCA	228
Ccnd1	mouse	GGGTGGGTTGGAAATGAAC<br>TCCTCTCCAAAATGCCAGAG	110
Gapdh	mouse	TGTCAGCAATGCATCCTGCA<br>CCGTTCAGCTCTGGGATGAC	240

Supplementary Table S3. Antibodies for immunoblotting and immunohistochemistry

protein	Catalog number, Company	dilution
β-catenin	C2206, Sigma	1:4000
c-MYC	clone 5605, Cell Signaling Technology	1:1000
Cyclin D1	clone 2926S, Cell Signaling Technology	1:2000
H3K27me3	07-449, Millipore/Upstate	1:1000
GAPDH	#49-1008, Invitrogen	1:20000
TLE1	clone M101, Santa Cruz Biotechnology	1:50
BCL2	clone mw-26, Santa Cruz Biotechnology	1:500
pan-CK	MAB3412, Millipore	1:1400
EMA	M0613, Dako	1:400
IgG-HRP	sc-2004, Santa Cruz Biotechnology	1:5000

Buffers

Mild lysis buffer - 10 mM Tris-HCl pH 8.1, 10 mM NaCl, 0.5% NP-40, proteinase inhibitors
Nuclear wash buffer - 50 mM Tris-HCl pH 8.0, 100 mM NaCl, 10 mM EDTA, proteinase inhibitors
Nuclear lysis buffer - 50 mM Tris-HCl pH 8.0, 100 mM NaCl, 10 mM EDTA, 1% SDS, proteinase inhibitors

Supplementary Table S4. Gene Expression Omnibus accession numbers for mouse tumors used in non-hierarchical clustering with synovial sarcomas with and without genetically stabilized β-catenin

Cancer Type	Reference	Parent Array SetGEO Accession	Sample GEO Accession
multiple myeloma	[1]	GDS2640	GSM160869
			GSM160880
			GSM160881
			GSM160882
			GSM160883
			GSM160884
papillary thyroid cancer	[2]	GSE10743	GSM271095
			GSM271096
			GSM271174
			GSM271175
acute myelogenous leukemia	[3]	GSE15195	GSM379298
			GSM379299
			GSM379300
invasive ductal breast carcinoma	[4]	GSE21444	GSM536027
			GSM536028
			GSM536029
			GSM536030
			GSM536031
colorectal carcinoma	[5]	GSE21576	GSM538813
			GSM538814
			GSM538815
			GSM538816
			GSM538817
pleiomorphic sarcoma NOS	[6]	GSE22841	GSM564575
			GSM564576
			GSM564577
			GSM564578
rhabdomyosarcoma			GSM564567
			GSM564568
			GSM564569

(*Continued*)

Cancer Type	Reference	Parent Array SetGEO Accession	Sample GEO Accession
			GSM564570
hepatocellular carcinoma	[7]	GSE26538	GSM652420
			GSM652421
			GSM652422
			GSM652423
			GSM652424
			GSM652425
squamous cell carcinoma	[8]	GSE29328	GSM855520
			GSM855521
			GSM855522
			GSM855523
lung carcinoma	[9]	GSE31013	GSM768531
			GSM768532
			GSM768533
			GSM768534
			GSM768535
			GSM768536
Burkitt lymphoma	[10]	GSE35219	GSM864016
			GSM864017
			GSM864018
			GSM864019
			GSM864020
			GSM864021
prostate carcinoma	[11]	GSE35247	GSM864529
			GSM864532
			GSM864533
			GSM864535
			GSM864536
			GSM864538
melanoma	[12]	GSE39984	GSM982953
			GSM982954
			GSM982955
			GSM982956
			GSM982957
			GSM982958

(*Continued*)

Cancer Type	Reference	Parent Array SetGEO Accession	Sample GEO Accession
pancreatic ductal adenocarcinoma	[13]	GSE40609	GSM997800
			GSM997803
			GSM997806
			GSM997813
			GSM997816
Kaposi sarcoma	[14]	GSE6482	GSM149228
			GSM149229
			GSM149230
osteosarcoma	[15]	GSE9460	GSM240288
			GSM240289
			GSM240291
			GSM240292
			GSM240295

REFERENCES

1. Carrasco DR, et al. The differentiation and stress response factor XBP-1 drives multiple myeloma pathogenesis. Cancer Cell. 2007; 11:349–60.
2. Burniat A, et al. Gene expression in RET/PTC3 and E7 transgenic mouse thyroids, RET/PTC3 but not E7 tumors are partial and transient models of human papillary thyroid cancers. Endocrinology. 2008; 149:5107–17.
3. Lo MC, et al. Combined gene expression and DNA occupancy profiling identifies potential therapeutic targets of t(8; 21) AML. Blood. 2012; 120:1473–84.
4. Kretschmer C, et al. Identification of early molecular markers for breast cancer. Mol Cancer. 2011; 10:15.
5. Deka J, et al. Bcl9/Bcl9l are critical for Wnt-mediated regulation of stem cell traits in colon epithelium and adenocarcinomas. Cancer Res. 2010; 70:6619–28.
6. Hettmer S, et al. Sarcomas induced in discrete subsets of prospectively isolated skeletal muscle cells. Proc Natl Acad Sci U S A. 2011; 108:20002–7.
7. Hoenerhoff MJ, et al. Global gene profiling of spontaneous hepatocellular carcinoma in B6C3F1 mice, similarities in the molecular landscape with human liver cancer. Toxicol Pathol. 2011; 39:678–99.
8. Schober M, Fuchs E. Tumor-initiating stem cells of squamous cell carcinomas and their control by TGF-beta and integrin/focal adhesion kinase (FAK) signaling. Proc Natl Acad Sci U S A. 2011; 108:10544–9.
9. Pandiri AR, et al. Differential transcriptomic analysis of spontaneous lung tumors in B6C3F1 mice, comparison to human non-small cell lung cancer. Toxicol Pathol. 2012; 40:1141–59.
10. Sander S, et al. Synergy between PI3K signaling and MYC in Burkitt lymphomagenesis. Cancer Cell. 2012; 22:167–79.
11. Ding Z, et al. Telomerase reactivation following telomere dysfunction yields murine prostate tumors with bone metastases. Cell. 2012; 148:896–907.
12. Kwong LN, et al. Oncogenic NRAS signaling differentially regulates survival and proliferation in melanoma. Nat Med. 2012; 18:1503–10.
13. Eser S, et al. Selective requirement of PI3K/PDK1 signaling for Kras oncogene-driven pancreatic cell plasticity and cancer. Cancer Cell. 2013; 23:406–20.
14. Mutlu AD, et al. *In vivo*-restricted and reversible malignancy induced by human herpesvirus-8 KSHV, a cell and animal model of virally induced Kaposi's sarcoma. Cancer Cell. 2007; 11:245–58.
15. Walkley CR, et al. Conditional mouse osteosarcoma, dependent on p53 loss and potentiated by loss of Rb, mimics the human disease. Genes Dev. 2008; 22:1662–76.
